# “When Rest Feels Wrong”: A Qualitative Study of Rest Intolerance Among Nursing Interns and Implications for Workforce Resilience

**DOI:** 10.1155/jonm/4480060

**Published:** 2026-06-15

**Authors:** Jingjing Cai, Furong Chen, Ying Xiong, Qihan Zhang, Liqun Zhou, Jiagen Xiang, Jiaying Li, Zengjie Ye

**Affiliations:** ^1^ School of Nursing, Guangzhou University of Chinese Medicine, Guangzhou, Guangdong, China, gzucm.edu.cn; ^2^ School of Nursing, Guangzhou Medical University, Guangzhou, Guangdong, China, gzhmc.edu.cn; ^3^ The First Affiliated Hospital, Guangzhou University of Chinese Medicine, Guangzhou, Guangdong, China, gzucm.edu.cn; ^4^ The Nethersole School of Nursing, Faculty of Medicine, The Chinese University of Hong Kong, Hong Kong SAR, China, cuhk.edu.hk

**Keywords:** nursing interns, phenomenological research, professional identity, rest intolerance, social comparison, transition shock, workforce resilience

## Abstract

**Objective:**

To explore the psychological experience and underlying mechanisms of “rest intolerance” among nursing interns.

**Background:**

Effective recovery is essential for preventing burnout and ensuring patient safety. However, nursing interns often experience “rest intolerance”, a paradoxical psychological state where resting induces distress rather than recovery. As interns represent the future pipeline of the nursing workforce, understanding this phenomenon is critical for managers aiming to build resilient teams and reduce early‐career attrition.

**Design:**

A qualitative interpretive phenomenological study.

**Methods:**

Twenty‐one nursing interns were recruited via purposive sampling. Data collected through semi‐structured interviews were analyzed using Colaizzi’s method.

**Results:**

Six themes and fourteen subthemes emerged. Participants described rest intolerance as involving four interrelated psychological experiences: (Theme 1) maladaptive cognitive ruminations (obsessive thinking and guilt regarding downtime); (Theme 2) toxic social comparison (viewing peers’ activity as a benchmark for self‐worth); (Theme 3) distorted professional identity (equating rest with laziness or lack of commitment); and (Theme 4) psychological inability to disengage (passive anxiety states). Participants attributed these states to two primary systemic stressors: (Theme 5) transitional shock and (Theme 6) task‐time resource imbalance.

**Conclusions:**

Interns suffer a “recovery deficit” associated with a cultural and psychological inability to accept rest, which may contribute to burnout and turnover.

**Implications for Nursing Management:**

Findings suggest that simply providing time off may be insufficient if organizational culture stigmatizes rest. Accordingly, nurse managers and educators could (1) move beyond resilience training to address “rest guilt” explicitly; (2) model healthy recovery behaviors to help dismantle “hustle culture” in clinical wards; and (3) structure internships to reduce the task‐time imbalance that appears to trigger cognitive dissonance during downtime.

**No Patient or Public Contribution:**

This research did not involve patient or public participation in its design, conduct, data collection, analysis, or manuscript preparation. No patients or members of the public were involved in the development of the research question, interpretation of results, or dissemination of findings.

## 1. Introduction

The sustainability of the global nursing workforce hinges on the successful transition of students into professional practice [1]. With the World Health Organization (WHO) projecting a global shortage of 4.6 million nurses by 2030 [2], and China’s nurse‐to‐population ratio (3.7 per 1000) remaining significantly below recommended standards [3–5], the retention of nursing interns is a critical priority. As the future pipeline of the workforce, interns face immense pressure to demonstrate competence. However, their ability to sustain this performance depends heavily on their ability to recover from work demands. While the physical necessity of rest is well‐understood, a paradoxical psychological phenomenon known as “rest intolerance” has emerged as a significant barrier to effective recovery and workforce resilience [6, 7].

“Rest shame,” sometimes summarized as “time sickness” in medicine, refers to an obsessive belief that time is constantly slipping away and is never sufficient [8]. In this paper, we use the term “rest intolerance” to describe this phenomenon: painful self‐conscious affect during rest, accompanied by feelings of defectiveness or moral failing [9]. It commonly manifests as intrusive, work‐related thoughts during downtime and negative emotions such as guilt, distress, and anxiety [10]. Importantly, it differs from general fatigue or burnout in its moral judgment of rest rather than exhaustion, from leisure guilt in its broader affective range and obsessive thinking and from anxiety disorders in its rest‐specific triggers and self‐conscious emotions such as shame and guilt [3]; these conceptual distinctions help prevent construct overlap and have been empirically supported [11]. Unlike general rumination, which is a broad response to distress, rest intolerance involves rest‐specific obsessive thinking about wasted time and moral self‐judgment [12]. Previous studies have shown that rest intolerance can negatively impact both mental and physical health across different populations, leading to serious outcomes such as reduced subjective well‐being, heightened stress levels, increased anxiety, and lower self‐rated health, particularly among college students facing significant academic stress. For nursing interns, this creates a dangerous cycle: the need for rest increases due to clinical demands, yet the psychological capacity to accept rest decreases, leading to heightened stress, sleep disorders, and reduced subjective well‐being [13, 14].

This phenomenon is particularly acute in high‐performance cultural contexts like China. Steeped in Confucian values that emphasize diligence and collectivism, Chinese education often frames rest as a sign of “falling behind” or a lack of motivation [15–17]. Consequently, nursing interns operate in an environment where downtime is stigmatized, and peer comparison is pervasive [18, 19]. When this cultural pressure intersects with “transition shock”, the disorientation experienced when moving from student to professional roles, interns may view rest as a professional weakness rather than a physiological necessity [17]. Unlike general student populations, nursing interns face the added weight of direct patient responsibility, ongoing clinical evaluation, and the high‐stakes transition from learner to practitioner. These factors amplify the perceived consequences of taking rest [20–22]. This cognitive dissonance creates a unique managerial challenge: providing time off is insufficient if the interns are psychologically unable to utilize it without guilt. Despite the implications for workforce retention and safety, empirical research on rest intolerance remains limited. While Wang et al. have developed scales to measure this construct quantitatively, there is a scarcity of qualitative inquiry describing the lived experience of this phenomenon [11]. Understanding the specific cognitive and environmental triggers of rest intolerance is essential for designing interventions that go beyond scheduling changes to address the underlying professional culture.

Therefore, this study adopted an interpretive phenomenological approach to explore how nursing interns experience rest intolerance and to identify the period‐specific stressors that fuel it. The findings aim to inform nursing educators and managers in developing targeted support systems that validate rest as a component of professional competence.

## 2. Methods

### 2.1. Design and Context

A key methodological principle of interpretive phenomenology is to explore and interpret the lived meanings of a phenomenon while acknowledging the researcher’s role in meaning‐making [23–25]. Specifically, we adopted a van Manen orientation, which legitimizes thematic abstraction, identification of experiential patterns, and visual modeling without claiming causal explanation [26]. This approach was selected because it encourages reflexivity on behalf of bracketing and utilizes researchers’ insights in meaning interpretation. The concept of rest intolerance in nursing interns is an understudied phenomenon with multiple dimensions; thus, interpretive phenomenology was chosen to gain an in‐depth understanding of participants’ lived experiences while allowing for interpretive synthesis. This study was reported following the Standards for Reporting Qualitative Research (SRQR) checklist. The completed checklist is provided as supporting information (available here). No artificial intelligence or large language models were used in any aspect of this study.

### 2.2. Researcher Characteristics and Reflexivity

Interviews were conducted by a female postgraduate nursing student with a prior 6 month internship experience in a tertiary hospital. Her experiential proximity facilitated rapport yet posed a risk of over‐identification; reflexivity was ensured through a contemporaneous reflective diary (documenting assumptions, decisions, and emotions), scheduled supervisory debriefs, and reflexive memos documenting assumptions and potential biases before and after each interview. During analysis meetings, reflexive memos were reviewed prior to coding to surface and critically examine assumptions, and they were revisited whenever interpretive disagreements arose, ensuring that participants’ voices remained central. To mitigate bias, field notes were triangulated with interview transcripts, and preliminary interpretations were discussed within the research team. Member checking was conducted with selected participants to confirm the accuracy of the synthesized themes.

### 2.3. Participants and Setting

Using purposive sampling [23, 27], we recruited undergraduate and postgraduate nursing interns from the Be Resilient to Nursing Career (BRNC) program [28–35]. This program is implemented at Guangzhou University of Chinese Medicine and Guangzhou Medical University, and the recruited interns can fully grasp the researchers’ conceptual understanding of rest intolerance. Inclusion criteria are (1) undergraduate or postgraduate nursing status; (2) being currently engaged in a clinical internship between October 2024 and July 2025; (3) ability and willingness to provide informed consent; and (4) sufficient fluency in Mandarin to participate in an in‐depth interview. Students who had withdrawn from the internship were excluded.

### 2.4. Ethical Considerations

This study obtained ethical approval from the Ethics Committee of the First Affiliated Hospital of Guangzhou University of Chinese Medicine (ZYYEC‐ERK [2020] 132). Consistent with the ethical principles outlined in the Declaration of Helsinki, all participants provided written informed consent after receiving a detailed verbal explanation of the study procedures and key objectives. All participants were further assured that their personal information would be treated with strict confidentiality and that their anonymity would be fully protected throughout the research process.

### 2.5. Procedure and Data Collection

Prior to the formal interviews, two nursing interns meeting the inclusion criteria were recruited for pilot interviews. Guiding questions for the interviews were developed based on existing literature and expert input from a PhD candidate with expertise in qualitative research (C.F.R.) and a clinical nursing education specialist (Z.L.Q.). This process aimed to verify question relevance, identify potential issues, and refine the research team’ s interview skills. Following the pilot interviews, the interview guide was revised iteratively by integrating pilot interview findings and experts’ ongoing supervisory feedback, leading to the finalization of the formal interview guide (Table 1).

**TABLE 1:** Interview guide questions.

1‐Can you describe your clinical nursing internship experience?
2‐During the internship period, what feelings do you have when resting?
3‐When resting or relaxing, do you worry that your peers are working harder than you or will surpass you?
4‐Do you have any intrusive thoughts when resting during the internship?
5‐What role do you believe rest plays in your internship?
6‐From a nursing intern’s perspective, how would you define rest intolerance? What are its core components?
7‐What factors do you consider to be the causes of rest intolerance?
8‐What strategies do you think could help avoid or alleviate rest intolerance?

To collect basic information about participants, a WeChat communication containing an invitation letter and a participant information form was sent to eligible individuals 3 days prior to the interviews. Upon receiving their responses, we confirmed the specific time and location of the interviews.

Following self‐introductions and the establishment of basic trust with participants, face‐to‐face semistructured interviews were conducted in a private room with only one researcher and one participant present per session. To build rapport, the interviewer (C.J.J.) conducted interview sessions using the final semistructured interview guide, each lasting approximately 20–30 min. All interviews were audio‐recorded following written informed consent. Exploratory, non‐leading prompts derived from the interview guide were used to elicit detailed, in‐depth accounts of rest intolerance during the internship. Participants were encouraged to speak freely without judgment or inducement, and the interviewer took brief field notes to document contextual details. Participants were provided with the opportunity to review and revise the transcripts prior to the initiation of data analysis, to ensure the accuracy of their perspectives.

Given the qualitative nature of this study, the sample size was not pre‐specified. Consequently, the number of participants was determined by the collected data. Data collection ceased upon reaching data saturation—defined as the point at which subsequent interviews failed to generate new insights related to the study’s focal concept of “rest intolerance”. Data analysis proceeded concurrently with data collection.

### 2.6. Data Processing and Analysis

Ultimately, data saturation was achieved after contacting and interviewing 21 consecutive participants. The analysis proceeded concurrently with data collection, guided by an interpretive phenomenological approach. Following each interview, audio recordings were transcribed verbatim, cross‐checked against the original recordings for accuracy, and imported into NVivo 15 for data management. We adapted Colaizzi’s seven‐step method as an analytic framework within an interpretive phenomenological orientation (1‐repeated immersion in the transcripts; 2‐extraction of statements relevant to rest intolerance and transition shock; 3‐formulation of meanings and initial codes; 4‐clustering codes into theme categories; 5‐development of detailed thematic descriptions; 6‐synthesis of the essential structure and core themes; 7‐participant validation) to enhance interpretive credibility. Coding was iterative and comparative. Two researchers (C.J.J. and C.F.R.) independently coded the first five transcripts (23.8% of the sample). They then compared coding and discussed discrepancies. All disagreements were resolved through team discussion, with a third researcher (Z.L.Q.) arbitrating when consensus could not be reached after two rounds of discussion. After establishing agreement on the initial set, the remaining transcripts were coded by C.J.J., while C.F.R. double‐coded four randomly selected transcripts (19.0% of the sample) to ensure consistency. Regular debriefing sessions were held, and an audit trail documented all coding decisions and thematic revisions.

### 2.7. Trustworthiness

To guarantee the data trustworthiness, the Guba and Lincoln criteria—encompassing credibility, dependability, confirmability, and transferability—were used [23, 36, 37]. Furthermore, to enhance data credibility, member checking was employed alongside the research team’s prolonged engagement with the research context. To ensure confirmability, we used internal peer debriefing (the research team reviewed coding and themes, resolving discrepancies through consensus) and external auditing (two independent qualitative researchers reviewed raw data, coding categories, and thematic development). To ensure data dependability, an audit trail was implemented. In the application of this methodology, the researcher retained the raw data, established categories, and identified initial themes throughout the entire research process. The transferability of the study additionally relied on the evaluation and validation of the findings by individuals operating within the same contextual environment. Devoting adequate time to the study and conducting face‐to‐face interactions with participants were other key factors that bolstered data credibility.

## 3. Results

Ultimately, data saturation was achieved after contacting and interviewing 21 consecutive nursing interns: 15 undergraduates and 6 postgraduates, aged 20–26 years (2 males, 19 females). Participants were recruited from multiple provinces across China, thereby enhancing the diversity of their training backgrounds (Table 2).

**TABLE 2:** Characteristics of participants (*n* = 21).

Participant	Sex	Age	Birthplace (province)	Educational level	Internship duration (months)	Clinical internship department (at interview)
1	Female	22	Guangdong	Undergraduate	4	Breast Surgery Department
2	Female	22	Heilongjiang	Undergraduate	4	Orthopedics Department
3	Female	21	Shandong	Undergraduate	5	Surgical Intensive Care Department
4	Female	22	Guangdong	Undergraduate	5	Cardiovascular Surgery Department
5	Female	22	Guangdong	Undergraduate	6	Emergency Department
6	Female	22	Hubei	Undergraduate	6	Gastroenterology Department
7	Female	22	Guangdong	Undergraduate	6	Otolaryngology Department
8	Male	22	Guangdong	Undergraduate	7	Nephrology Department
9	Female	22	Xinjiang	Undergraduate	7	Intensive Care Department
10	Female	22	Guangdong	Undergraduate	7	Operating Room Department
11	Female	21	Guangdong	Undergraduate	8	Gynecology Department
12	Female	24	Xinjiang	Undergraduate	8	Cardiology Department
13	Female	22	Hubei	Undergraduate	9	Dermatology and Rheumatology Department
14	Female	22	Jilin	Undergraduate	9	Emergency Department
15	Female	20	Guangdong	Undergraduate	9	Blood Purification Department
16	Female	26	Henan	Postgraduate	8	Obstetrics Department
17	Male	26	Guangdong	Postgraduate	9	Gynecology Department
18	Female	21	Hunan	Postgraduate	11	Gynecology Department
19	Female	24	Guangdong	Postgraduate	11	Endocrinology Department
20	Female	24	Anhui	Postgraduate	12	Gynecology Department
21	Female	24	Guangdong	Postgraduate	13	Breast Surgery Department

**FIGURE 1:**
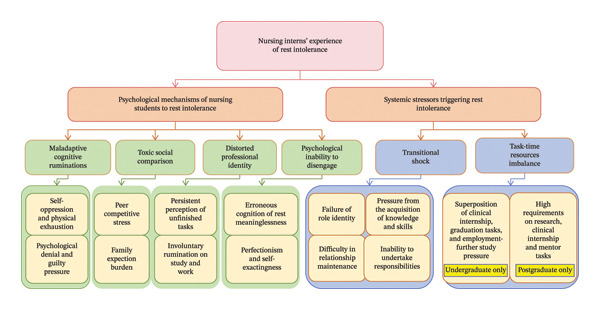
Thematic map of experiential patterns of rest intolerance among nursing interns.

Through in‐depth analysis, six themes were identified: maladaptive cognitive ruminations, toxic social comparison, distorted professional identity, psychological inability to disengage, transitional shock, and task‐time resource imbalance. These themes illustrate participants’ accounts of the psychological patterns underlying rest intolerance and the systemic stressors as reported by participants, with the latter two described as the primary triggers for the former four. Figure 1 presents a thematic map of experiential patterns illustrating the relationships among the six themes as described by participants, with Themes 5 and 6 positioned as the primary systemic stressors triggering Themes 1–4.

To avoid conceptual overlap among themes, we clarify the distinctions as follows: Theme 1 captures affective and physiological responses specifically during rest (guilt, self‐oppression); Theme 3 reflects identity‐driven pressure wherein rest is perceived as a violation of the professional nursing role; and Theme 4 represents general cognitive schemas “rest is a waste of time” and perfectionism not tied to professional identity. Each theme includes subcategories, supported by participant quotations detailed below.

### 3.1. Psychological Mechanisms of Nursing Students to Rest Intolerance

#### 3.1.1. Theme 1. Maladaptive Cognitive Ruminations (Obsessive Thinking and Guilt Regarding Downtime)

Confronted with the impact of rest intolerance, numerous nursing interns highlighted significant changes in both their physical and psychological patterns and preferences. Physiologically, this phenomenon is characterized by self‐imposed pressure, anorexia, and sleep disturbances, alongside other related manifestations. Psychologically, affected nursing interns concurrently exhibit emotional distress, including self‐denial, guilt, and other negative emotional states.

##### 3.1.1.1. Self‐Oppression and Physical Exhaustion

Fourteen/twenty‐one of the sample highlighted “overworking” during designated rest periods, sacrificing recovery time to complete tasks and forming a pattern of “only feeling at ease when not resting”. This was associated with sleep disturbances, changes in appetite, and somatic arousal (e.g., palpitations).

*“Instead of going to sleep after my night shift, I shadowed the consultant performing a urinary catheterisation on the ward to practise the technique. I kept thinking, ‘Every extra minute of learning will ease future anxiety’.”* (Participant 1)

*“Suddenly, while I was resting, I had palpitations—my heart was racing so fast I couldn′t catch my breath. Even when I did fall asleep, I kept having recurring dreams where I was rushing to finish a project report.”* (Participant 12)

##### 3.1.1.2. Psychological Denial and Guilty Pressure

Seventeen/twenty‐one nursing interns reported anxiety, guilt, irritability, and episodic emotional outbursts during rest; the more they rested, the more psychological discomfort and self‐denial they reported.

*“I’m consistently plagued by guilt and regret for not completing my tasks, convinced that I’m constantly wasting time and feeling irritable. I also find myself avoiding social interaction, yet when I’m alone, I’m more prone to mental exhaustion and sadness.”* (Participant 13)

“I feel that my emotions are far more volatile since starting the placement, with significant highs and lows. I also have a persistent sense of mental agitation.” (Participant 1)

#### 3.1.2. Theme 2. Toxic Social Comparison (Viewing Peers’ Activity as a Benchmark for Self‐Worth)

Respondents commonly described managing parallel demands, academic tasks, internship duties, job preparation, and family expectations within a clinical culture that can be critical of interns. These pressures appeared to foster social comparison with patients, instructors, and peers and were associated with intensified guilt and rest intolerance.

##### 3.1.2.1. Peer Competitive Stress

Amid a stressful clinical environment, insufficient instructor guidance, prejudice against interns, and traditional Chinese cultural norms emphasizing unquestioned pursuit of excellence and diligence, 16/21 participants reported that they had gradually developed comparative stress from monitoring their peers’ progress, ultimately leading to self‐doubt.

“I devote all my spare time to learning and practising practical skills, to avoid being compared unfavourably to the other trainees by my preceptor. My goal is to be seen as the student with the most proficient hands‐on abilities in her eyes.” (Participant 20)

*“Knowing that my classmates have already met the graduation requirements and published papers, I’m filled with guilt whenever I take time to relax. I think, ‘they are making progress while I’m slacking off; resting is the same as falling behind’.”* (Participant 16)

##### 3.1.2.2. Family Expectation Burden

Eleven/twenty‐one nursing interns stated that they were frequently questioned by patients and their families. Burdened by family expectations—including the pressure to support their families immediately after graduation—and constant comparisons with other family members, they focused exclusively on advancing without rest, thereby developing the psychological experience of rest intolerance.

“My sister has a career that meets my parents’ expectations, and my brother is at a more prestigious university. I feel that studying nursing is a let‐down to the family.” (Participant 4)

“All my siblings secured postgraduate places ‐ I feel like a failure for not having succeeded. Although my family have never said it outright, I’m under immense pressure, and feel I must study every day without daring to take a break.” (Participant 14)

#### 3.1.3. Theme 3. Distorted Professional Identity (Equating Rest With Laziness or Lack of Commitment)

Competing academic, clinical, and employment tasks often remained mentally unresolved, prompting ruminative monitoring and intrusive task related thoughts during rest. Consequently, 19/21 respondents struggled to disengage from work‐related stress.

##### 3.1.3.1. Persistent Perception of Unfinished Tasks

Sixteen/twenty‐one participants indicated they were plagued by unresolved tasks, closely monitoring deadlines for academic requirements, clinical internships, research projects, and job recruitment. Over time, even in the absence of pending tasks, they became trapped in an emotional cycle of persistent anxiety and persistently felt there were unresolved tasks.

“The night before our group meeting, even when I was trying to rest, I was plagued by the thought that my preparations were insufficient and could be improved.” (Participant 21)

“I find it impossible to truly switch off. Even when one task is completed, I’m constantly aware of many more waiting for me. I’ve been in a persistent state of anxiety and find myself caught in cycles of obsessive rumination.” (Participant 1)

##### 3.1.3.2. Involuntary Rumination on Study and Work

Fourteen/twenty‐one participants mentioned they struggled to cease ruminating on work and study during rest, as work and study‐related concerns invaded their thoughts and plunged them into anxiety and self‐blame.

“Even when resting in my room or spending time off‐campus, I found myself constantly ruminating about ward matters and dwelling on negative experiences.” (Participant 8)

“While having a meal with classmates, seeing a message from my supervisor made me feel guilty for taking a break, and I immediately wanted to finish eating quickly and return to my research project.” (Participant 16)

#### 3.1.4. Theme 4. Psychological Inability to Disengage (Passive Anxiety States)

Shaped by traditional Eastern educational values and modern self‐development ideals, two erroneous cognitive patterns emerge: the belief that “rest is a waste of time” and the pursuit of extreme perfectionism. Many nursing interns indicated that they had gradually encountered such experiences during their placements.

##### 3.1.4.1. Erroneous Cognition of Rest Meaninglessness

Fourteen/twenty‐one interns affirmed the belief that “rest is a waste of time,” equating rest with laziness or insufficient diligence. This belief further reinforces a mindset that pits rest against progress, leading to significant encroachment of rest time by work and study commitments. Poor rest quality results in inadequate energy and suboptimal task performance, perpetuating a vicious cycle in which they have even less time for rest.

“I feel uneasy and agitated if I rest for too long, as I believe I have overindulged in leisure. Guided by the principle of ‘diligence and perseverance’, I view rest and recreation as fundamentally improper.” (Participant 10)

“Whenever I try to rest, I don′t feel content but I know I ought to take some time off. Afterwards, I’m filled with regret for not having used that time to study, which leaves me feeling distressed and guilty, trapping me in a cycle of inner conflict.” (Participant 14)

##### 3.1.4.2. Perfectionism and Self‐Exactingness

Driven by their admiration for excellence and superiority in Eastern culture, 9/21 participants found themselves trapped in the mental inertia of the imperative of “perfectionism” and “high self‐expectations”. They thus constantly cut back on their rest time and energy, striving to become “excellent individuals” as defined by universal values.

“I want to do things well so as not to let down my supervisor’s guidance. It would be unacceptable to present him with substandard work. I hold myself to very high standards: it’s an all‐or‐nothing approach for me, and I never handle tasks perfunctorily.” (Participant 6)

“My desire isn’t just to be on par with others, but to be demonstrably better than average. That is the root of my anxiety. I remain in a constant state of tension, going to the library every day without fail. I feel intensely anxious if I′m not studying, even during the exam period itself.” (Participant 1)

### 3.2. Systemic Stressors Triggering Rest Intolerance

#### 3.2.1. Theme 5. Transitional Shock

Amid transitional shock, 19/21 nursing interns faced multifaceted pressures arising from role transition, relationship adjustment, knowledge and skill acquisition, and responsibility assumption. These contextual and phase‐specific pressures persistently contributed to rest intolerance and self‐reproach during rest periods.

##### 3.2.1.1. Failure of Role Identity

Eight/twenty‐one nursing interns reported limited autonomy and a mismatch between idealized nursing role expectations and routine task reality during transition. Role ambiguity and competence anxiety led to overcompensation by prioritizing work and study. They perceived rest as a threat to role validation, fearing downtime exacerbates feelings of inadequacy or unmet role expectations. This fueled guilt, shame, and the belief that “rest equals incompetence,” distorting perceptions of rest and causing persistent rest reluctance and rest intolerance.

“I still perceive myself very much as a student. When interacting with patients, I feel particularly conflicted about whether I should be communicating from the position of a ‘learner’ or that of a ‘nurse’.” (Participant 3)

“Moving from the classroom into clinical practice, I haven’t yet adapted to the transition of ‘being responsible for patients’. I constantly worry that my understanding of the role is incorrect and will cause issues. Consequently, I devote a great deal of my personal time to studying, hoping to become a competent nurse as soon as possible.” (Participant 5)

##### 3.2.1.2. Difficulty in Relationship Maintenance

Seven/twenty‐one participants described that difficulties in relationship maintenance—including interpersonal adjustment challenges and communication strain with colleagues or supervisors during internships—are associated with rest intolerance. Interns perceived rest as wasted time or a risk of neglecting relationships, thereby fostering guilt and anxiety. This led them to sacrifice rest for relationship building, distorting their perception of rest’s value and ultimately contributing to persistent rest intolerance.

“When communicating with my preceptor, I’m always concerned about disturbing her with too many questions. I often don’t dare to voice my confusions, which has resulted in a consistently distant relationship between us. Sometimes, these thoughts still occupy my mind after work.” (Participant 12)

“My colleagues’ established routines make it difficult for me to integrate, leaving me feeling isolated. The demanding placement leaves little time for personal relationships, and I sometimes take out my frustrations on them. This affects my sleep and overall well‐being.” (Participant 20)

##### 3.2.1.3. Pressure From the Acquisition of Knowledge and Skills

Twelve/twenty‐one participating interns highlighted that they were required to master extensive professional knowledge and skills during internships. Consequently, interns viewed excessive rest as hindering skill acquisition, inducing anxiety and self‐reproach. Prioritizing learning over rest, they developed a skewed perception of rest, which ultimately led to persistent rest intolerance.

“There are so many practical procedures to master. I must have practised intravenous puncture dozens of times, but my hands still tremble. I worry that I’ll never properly learn it and will be asked to leave.” (Participant 15)

“My preceptor’s standards are exceptionally high; every procedure must be performed with precision. I consistently fail to meet the mark, and the pressure is so immense that I have trouble sleeping.” (Participant 4)

##### 3.2.1.4. Inability to Undertake Responsibilities

Ninth/fifteen undergraduate interns stated that perceptions of limited fault tolerance in clinical practice heightened fear of negative consequences and sustained hypervigilance outside of work hours. Furthermore, as their sense of responsibility for future professional development was being formed, nursing interns’ failure to meet their professional obligations plunged them into self‐reproach and anxiety, exacerbating symptoms of rest intolerance. Conversely, these symptoms can also induce responsibility avoidance, perpetuating a vicious cycle.

*“When asked to take sole responsibility for patients, I become particularly apprehensive about making mistakes. Even with routine tasks like fluid replacement, I find myself checking and rechecking everything. This constant vigilance leaves me perpetually tense, unable to allow myself even a moment’s respite.”* (Participant 12)

“While textbooks emphasize strict protocols like ‘three checks and seven verifications’, my mentor advises adjusting the angle based on depth for elderly patients with fragile veins. Struggling with this technique after unsuccessful attempts, I blame myself and think ‘Textbook knowledge is too rigid—I need to be more adaptable.’” (Participant 7)

#### 3.2.2. Theme 6. Task‐Time Resource Imbalance

Eighteen/twenty‐one undergraduate and postgraduate nursing interns faced multifaceted demands encompassing clinical practice pressure, academic research, further study, and job preparation. To meet these demands, they often sacrifice rest—staying up late to organize notes or practice with models. This imbalance induces persistent tension, diminished efficiency, and negative emotions, disrupting rest patterns and perpetuating rest intolerance cycles, ultimately compromising internship quality and personal well‐being.

##### 3.2.2.1. Superposition of Clinical Internship, Graduation Tasks, and Employment‐Further Study Pressure (Undergraduate Only)

Twelve/fifteen undergraduate nursing interns confront multifaceted pressures encompassing clinical practice, nursing license exam preparation, and job search—inducing time constraints and anxiety. They proactively sacrifice rest to prioritize tasks, but insufficient sleep and mental exhaustion diminish efficiency and elicit frustration, reinforcing the belief that “one must work harder and cannot rest.” This perpetuates a vicious cycle: task overload induces rest refusal, which further reinforces rest reluctance and ultimately consolidates into rest intolerance.

“Seeing peers constantly upskilling makes even a short break feel like falling behind, and the resulting anxiety disrupts my sleep. I’m terrified this relentless pace will lead to failure in everything.” (Participant 9)

“Juggling full ward duties means I can only revise late at night, leaving me exhausted the next day and affecting my placement performance. I try to squeeze in question practice during breaks or after shifts, but I’m often too tired to focus. It feels like a draining cycle that’s hard to sustain.” (Participant 15)

##### 3.2.2.2. High Requirements on Research, Clinical Internship and Mentor Tasks (Postgraduate Only)

Five/six postgraduate nursing interns navigated overlapping clinical schedules, research deadlines, and supervisory demands, resulting in chronic time conflicts. These conflicts compromised work‐rest balance and intensified guilt during leisure, rendering rest “unjustifiable”.

“The department runs on a mixed rota of clinical and academic shifts. Between placements, admin work, and pushing forward with my own research, I’m constantly busy—even catching up on missed shifts after time off. While it keeps me on my toes, the pressure leaves me genuinely anxious and affects my sleep and daily routine.” (Participant 17)

“Balancing the haemodialysis unit and project work, I also faced patient refusals in radiology. Work consumes my thoughts constantly, leaving me unable to switch off. Between my supervisor’s demands and my peers’ progress, any rest is overshadowed by guilt and anxiety about falling behind.” (Participant 16)

## 4. Discussion

Our study aims to explore the psychological experiences of rest intolerance among nursing interns, identify the triggering stressors, and provide empirical insights for the formulation of targeted support and intervention strategies. The six themes and fourteen subthemes identified in this study include two core dimensions: (a) psychological mechanisms—Theme 1 (maladaptive cognitive ruminations), Theme 2 (toxic social comparison), Theme 3 (distorted professional identity), Theme 4 (psychological inability to disengage) and (b) primary systemic stressors—Theme 5 (transitional shock) and Theme 6 (task‐time resource imbalance).

Nursing interns commonly experienced dual pressures of physical exhaustion and psychological distress during internship, aligning with evidence of overextension of physical and mental resources during their transition periods [38]. Their prior work identified the negative feelings as a core dimension associated with rest intolerance among nursing interns. Meanwhile, a study by Del Prato et al. showed that current support for nursing interns mostly focused on a single dimension, lacking collaborative intervention in physical and psychological aspects and limiting their effectiveness [39]. Our results suggest the need for a dual‐domain approach that addresses (a) physiological restoration and (b) psychological regulation. On the physical side, incorporating occupational health content (e.g., fatigue management, sleep hygiene, and microbreak protocols) may improve recognition of early physiological warning signals and reduce cumulative strain [40]. On the psychological side, brief mindfulness protocols can reduce anxiety [41], and cognitive‐behavioral strategies can challenge maladaptive beliefs such as “rest is a waste of time” [42, 43].

Beyond affective distress, in the Chinese cultural environment, social comparison, obsessive thinking, and cognitive bias were further described as contributing to rest intolerance. First, participants reported a tendency to engage in social comparison while resting, focusing on whether their peers continued working. Against the backdrop of intense educational competition in China [44], when peers choose to continue working during vacation periods, these individuals experience heightened rest‐related shame. This shame, which appeared to amplify negative emotions associated with rest [45], further strengthened their avoidance of rest as they strive to align with peers’ work‐oriented behaviors amid competitive pressures [46]. Second, this cultural pressure manifested as obsessive rumination. Participants shared an inability to mentally disconnect, experiencing intrusive, uncontrollable thoughts about deadlines even when physically away from the ward [47]. This persistent cognitive arousal appeared to disrupt their recovery process, suggesting a self‐perpetuating loop in which the fear of falling behind seemed to prevent the very rest needed to maintain performance [48]. Cognitive bias—equating rest with laziness and effort with moral worth—appears to be a proximal driver of rest intolerance, likely shaped by family socialization, school norms, and professional culture [49]. The notion of “no pain, no gain” is deeply embedded in Chinese cultural values [50]. Participants’ accounts indicated a tendency to hold a stronger belief in this principle, fostering a rest‐avoidant mindset and a negative predisposition toward rest. Such cognitive tendencies may reinforce their reluctance to engage in restorative rest, aligning with the core manifestations of rest intolerance [51].

Our study extends Duchscher’s theory of Transition Shock [51] by identifying it as a primary systemic catalyst for rest intolerance [52]. While transition shock typically describes the confusion and anxiety of role adaptation [53], our findings indicate that interns utilize “rest avoidance” as a maladaptive coping mechanism. Facing the “theory‐practice gap” and unfamiliar clinical responsibilities [54], interns equated continuous work with effective role adaptation. Rest is viewed as wasteful or indicative of incompetence, triggering guilt and anxiety that constitute core features of rest intolerance. Physical exhaustion from transition shock reinforced rest avoidance, creating a vicious cycle [55, 56]. Transitional pressure exacerbates rest intolerance, and insufficient rest in turn amplifies transition shock. Factors such as professional satisfaction and prejob training act as moderators in this relationship [53], suggesting that interventions must target this cycle early. Integrating “rest health” education into school–hospital collaborative training is therefore critical to breaking this feedback loop before it creates entrenched burnout [57].

The “Multitasking Burden” was described as a critical period‐specific driver of rest intolerance, particularly for postgraduate interns balancing the dual demands of clinical practice and academic research. Confronted with conflicting clinical shifts and thesis deadlines, these interns reframed rest not as a necessity but as an obstruction to their goals [58]. This scarcity mindset triggered the core manifestations of rest intolerance—guilt and anxiety—with downtime perceived as a direct threat to performance. While standard time‐management literature emphasizes prioritization for efficiency [59], our findings suggest these tools play a deeper psychological role. Interventions such as the four‐quadrant method and flexible clinical rostering during thesis windows may do more than organize time; participants’ accounts suggested they function as psychological scaffolds. By structurally defining “work time” versus “rest time” through weekly microgoals, managers may help interns regain a sense of control, reducing the cognitive overwhelm that fuels rest avoidance [10]. Thus, integrating rest‐health awareness into task management may be not merely an organizational strategy, but a potentially crucial intervention for preserving the mental resilience of the nursing workforce [60].

### 4.1. Limitations of Study

Several limitations of this study should be noted. First, participants were recruited exclusively from hospitals in Guangzhou, a tier‐one city. The findings may therefore reflect this specific high‐pressure environment and may not capture experiences from rural or less developed regions. Second, the sample was homogeneous in educational level (all from a baccalaureate program) and all participants came from the BRNC resilience‐focused program, which may have sensitized them to rest‐related concepts and limited diversity in experiential perspectives. Third, the sample was predominantly female, which limits our ability to explore potential gender differences in rest intolerance. Future research should include more diverse educational backgrounds, nonresilience‐focused programs, and male participants to capture a broader range of experiential profiles, including cases that may not align with the patterns identified here.

## 5. Conclusion

This research suggests that interns are caught in a paradox where the structural demands of the internship deplete their resources, while the professional culture psychologically prohibits them from recovering. Addressing this requires a paradigm shift: nursing management might need to move beyond simply providing time off to actively cultivating a professional environment that validates recovery as an essential component of clinical competence and patient safety.

## 6. Implications for Nursing Practice

To mitigate rest intolerance and facilitate a smoother transition to practice, nursing educators and clinical managers should adopt a collaborative approach that moves beyond simple time management. Educators can integrate “rest hygiene” into pre‐internship curricula, helping students reframe rest as a professional competency rather than a sign of laziness. Targeted interventions, such as workshops on transition shock and cognitive‐behavioral strategies, may help interns deconstruct the “guilt‐rest” cycle and reduce obsessive work‐related rumination.

On the clinical side, managers play a pivotal role in alleviating the task‐time imbalance that triggers rest intolerance. Rather than relying on detailed checklists or lengthy orientations, the core strategy is implementing flexible rostering during high‐pressure academic periods, combined with protected meal breaks and public recognition of healthy boundaries. Managers should actively cultivate psychological safety by modeling healthy work‐life boundaries, taking breaks, avoiding after‐hours work messages, and affirming intern rest, while also validating rest as essential for patient safety. By fostering active listening, safe dialogue, and nonjudgmental guidance from preceptors, clinical institutions can help dismantle the “hustle culture” in wards. These approaches ensure that interns feel empowered to utilize their downtime for genuine recovery.

## Author Contributions

Jingjing Cai: conceptualization, investigation, data curation, software, and writing–original draft. Furong Chen: conceptualization, methodology, and results interpretation. Ying Xiong and Qihan Zhang: data curation and writing revision. Liqun Zhou: conceptualization, methodology, and interview guide design. Zengjie Ye, Jiaying Li, and Jiagen Xiang: supervision, resources, and writing–review and editing.

## Funding

This study was supported by the Cultivation of Guangdong College Students’ Scientific and Technological Innovation, No. pdjh2023b0131.

## Conflicts of Interest

The authors declare no conflicts of interest.

## Supporting Information

Additional supporting information can be found online in the Supporting Information section.

## Supporting information


**Supporting Information** The SRQR Reporting Guideline Checklist (SRQR‐Reporting Guideline Checklist.pdf) is provided as supporting information.

## Data Availability

The data that support the findings of this study are available from the corresponding authors upon reasonable request.
